# Filial Maturity, Resolution of a Parent’s Disease, and Well-Being in Offspring of Parents Diagnosed with Alzheimer’s Disease

**DOI:** 10.3390/ijerph20010761

**Published:** 2022-12-31

**Authors:** Alon Goldberg

**Affiliations:** Department of Education, Tel-Hai College, Upper Galilee 12210, Israel; alongol@telhai.ac.il

**Keywords:** Alzheimer’s Disease, filial maturity, resolution of disease, well-being

## Abstract

Background: Alzheimer’s Disease (AD) is one of the most common forms of dementia. However, research dealing with the experience of adult children of a parent diagnosed with AD, regardless of whether the offspring is a caregiver, is not well developed. Objective: The current research is a cross-sectional study that examines the associations between filial maturity, offspring’s coming to terms with their parent’s AD, and the well-being of the offspring. Method: one hundred and forty Israeli adult children of parents with AD participated in the study and completed self-report questionnaires assessing their filial maturity, resolution of their parent’s diagnosis with AD, the adult children’s well-being, and the severity of the parent’s AD according neurologist’s report.Results: Results showed that higher resolution of the parent’s disease was positively associated with well-being. In addition, filial maturity was negatively associated with resolution of the parent’s disease, and resolution of the parent’s disease mediated the association between filial maturity and well-being. Conclusion: Resolution of a parent’s AD is highly challenging for offspring with high filial maturity, and the lack of resolution affects their well-being. Offering prolonged emotional support for offspring of parents diagnosed with AD may improve their ability to integrate the new reality into their lives and foster their well-being.

## 1. Introduction

Alzheimer’s Disease (AD) is one of the most common forms of dementia, constituting 60–80% of the 55 million dementia cases diagnosed worldwide [1]. AD’s symptoms are memory loss; difficulties with thinking and reasoning, decision making, and performing daily tasks; hallucinations; and frailty. The neurodegenerative process involved in AD also may cause personality changes. Further, most AD patients suffer from comorbidities (e.g., diabetes, cardiovascular disease, hearing and vision loss), which affect the clinical management of the disease and are associated with poor prognoses for the patients. Most persons with AD are generally 65 or older and live four to eight years after diagnosis, although there is evidence that some may live up to 20 years after diagnosis, and are cared for by a family member (i.e., spouse or adult child) [1]. As the disease progresses, the caregiver experiences predeath grief as a response to the multiple and continuous losses involved [2]. Losses of roles, relationships, and functions occur over a prolonged period before the death of the patient [3], and the caregiver witnessing the cognitive, physical, emotional, and behavioral regression of the spouse or parent suffers their own loss of the relationship they had and what could have been, as well as their own freedom [4], which may further affect the caregiver’s health and well-being [5]. The caregiver’s loss may be ambiguous, as the person with AD is physically present but increasingly mentally absent, which extends the sense of loss of the relationship the caregiver and the sick family member had before [6]. Blandin and Pepin [2] have suggested a unique three-stage model of grief for a better understanding of predeath grief of an individual with a significant family member with dementia: (1) Separation—acknowledgment of loss; (2) Liminality—experiencing ambiguous and difficult thoughts and feelings between the first and third stages of grief, although tolerating those thoughts and feelings may clarify the loss and contribute to adaptation to the new situation; and (3) Re-emergence—acceptance and acknowledgment of the situation and understanding its consequences.

There is substantial research focused on the well-being of family member caregivers (e.g., [2,7]). However, offspring of parents diagnosed with a neurodegenerative disease may experience a sense of continuous loss and may enter a predeath grief process regardless of whether the offspring are the primary caregivers [8]. Yet, little attention has been paid to offspring of parents diagnosed with neurodegenerative diseases who are not their primary caregivers; extending the research in this area to those individuals is necessary to understand the adult child’s experiences, well-being, coping strategies, and outcomes as might be expressed in their everyday life (i.e., marital relationships, parenting, etc.). The aim of the current study is to explore the association of these offspring’s filial maturity with their resolution of their parent’s diagnosis with AD and with the offspring’s well-being.

### 1.1. Filial Maturity

Blenkner [9] coined the term “filial maturity” to refer to the adult child’s perception of the parent as a reciprocal person with their own history, needs, and faults. Blenkner [9] described filial maturity as a significant developmental task in the relationship between adult children and parents, whereby the relationships between adolescents and parents end and a mature adult perception of the parent–child relationship begins. According to Blenkner [9], adult children start to see their parents’ weaknesses, which may bring a period of filial conflicts, as relationships are dealt with and eventually a new filial role is achieved. Filial maturity is reached when the adult child accomplishes the developmental task and succeeds in creating the new filial role. Marcoen [10] emphasizes filial maturity as a “dynamic state of continuous, successful coping with the normative task of parent care in middle-aged adult children” (p. 127), focusing on the obligation of adult children to provide care for their aging parents. On the other hand, Nydegger [11] considers filial maturity to be a developmental process that depends on the quality of the child’s interactions with the parents and begins in the child’s adolescence and finally matures in adulthood as the adult child’s distancing from the parent enables the child to develop a more objective perception of the relationship with the parent, and the adult child’s life enables her or him to better comprehend the parent’s world, life choices, and faults. These two conceptualizations of filial maturity were empirically tested and confirmed that filial maturity may relieve adult children’s difficulties with taking care of their increasingly aging and dependent parents [12,13]. Hence, the hypotheses of the current study suggest that offspring’s filial maturity might contribute to the resolution of their parent’s AD and eventually to the offspring’s well-being.

### 1.2. Offspring’s Resolution of Their Parent’s Disease

Bowlby [14] suggested that feelings of loss are inevitable and noticed that the reaction of an adult to the loss of a significant other, such as a spouse or a parent, resembles an infant’s reaction to separation from a parent. According to Bowlby [14,15], attachment relationships between the child and the parent are typically molded from birth through infancy and serve the person as an inner resource to regulate distress, by maintaining proximity to a significant other who provides comfort and relief. Attachment relationships between a child and his or her parent face changes throughout life and thus might be threatened [14]. The example that applies here (as in [8]) is when a parent is diagnosed with a life-threatening disease and the offspring experiences loss of the parent, anticipatory mourning, and predeath grief [16]. The offspring feels attachment needs for the parent, and when those are not met, emotional distress increases and the mourning process begins. At the end of a successful process, the child’s representations of the parent prior to the diagnosis must be adjusted to the new reality and integrated with the representations of the new parent post-diagnosis [8,17]. In the case of difficulties with the grief process and coming to terms with the parent’s disease, the individual is preoccupied with negative emotions and thoughts about the loss, or exhibits detached and distant behavior, while discrepancies between the offspring’s perceptions of the parent before and after the diagnosis still exist [8,14].

### 1.3. The Current Study

The current study examined the relationships between offspring’s filial maturity, their resolution of a diagnosis of a parent with AD, and well-being. In line with past literature regarding resolution of the loss and new representations and relationship with a parent diagnosed with a severe disease, I hypothesized, similar to [8], that:

**H1.**  *Offspring’s resolution of parent’s AD diagnosis will be positively associated with offspring’s higher well-being.*

In line with past literature that indicated that filial maturity may relieve adult children’s difficulties with taking care of their increasingly aging and dependent parents [12,13], I hypothesized that filial maturity is a resource of the offspring to cope and adjust to the new reality following the diagnosis of a parent with AD, and thus it would be associated with the resolution of their parent’s AD and with the offspring’s well-being.

**H2.**  *Offspring’s filial maturity will be positively associated with a better resolution of their parent’s AD diagnosis.*

**H3.**  *Offspring’s filial maturity will be positively associated with their higher well-being*.

I also explored whether filial maturity mediated the associations between offspring’s resolution of their parents’ AD diagnosis and the offspring’s well-being.

## 2. Method

### 2.1. Participants

Participants were 140 Israeli adult children of parents with AD. As shown in Table 1, the participants were close to 50 years old on average (*SD* = 10.67), 85.7% were female, 74.3% were married, 78.6% had a graduate or professional education, and 82.8% reported an average or above average income (average monthly income in Israel is approximately $2500) [18]. Parents diagnosed with AD were 80.41 years old on average (*SD* = 7.06); the average number of years since they got diagnosed was 4.56 years (*SD* = 3.30), at varying degrees of severity; 65.7% of parents were female; 45.7% were married and 40% were widowed; and 80% lived close to their adult child.

### 2.2. Procedure

Data were collected in a manner consistent with ethical standards for the treatment of human subjects, as part of a broader research study examining offspring’s resolution of their parents’ diseases. For more details regarding the research procedure, please see Goldberg [8].

To collect data for this part of the research, participants were asked to fill out the following questionnaires: Demographic questionnaire, an adapted RDQ [8], the Louvain Filial Maturity Scale (LFMS-A), and the WHO-5 Well-Being Index. Participants were also asked to report on the last MMSE score given their parent by their treating neurologist. Participants were informed that their anonymity would be preserved throughout the study and that they had the right to discontinue participation at any time. Roughly 25% of participants did not complete the questionnaires and hence were dropped from the study.

### 2.3. Instruments

#### 2.3.1. Demographic Questionnaire

Demographic details about offspring participants were collected compatible with Goldberg [8].

#### 2.3.2. The Louvain Filial Maturity Scale (LFMS)

The Louvain Filial Maturity Scale [10] is an 81-item self-report questionnaire that assesses the adult child and parent relationship, using a 7-point Likert scale ranging from 1 (“totally disagree”) to 7 (“totally agree”) on seven factors: filial love, filial obligation, filial autonomy, filial helpfulness, filial help, parental consideration, and family solidarity. Means score were calculated for each factor, with higher scores representing better filial maturity. Internal consistency in the current research ranged from 0.68 to 0.96. The filial autonomy factor was not used in the study due to its low internal consistency (0.46).

#### 2.3.3. Reaction to Diagnosis Questionnaire (RDQ) [19]

This 42-item self-report scale was originally developed to assess parents’ resolution of their child’s diagnosis and was adapted to assess offspring’s resolution with their parents’ disease [8]. In a sample of offspring of a parent diagnosed with Parkinson disease, it was found that offspring’s resolution with their parents’ disease was positively correlated with the offspring’s well-being and a better attachment with the parent [8]. Internal consistency in the current sample was 0.86.

#### 2.3.4. WHO-5 Well-Being Index

The WHO-5 is a self-report questionnaire [20,21,22], using five items to assess a person’s well-being over the last two weeks on a 6-point Likert scale from 0 (*not present*) to 5 (*constantly present*). For more details, see Goldberg [8]. Internal consistency in the current sample was 0.88.

#### 2.3.5. Mini-Mental State Examination (MMSE) [23]

The MMSE is a well-validated and widely used assessment of global cognitive impairment, using items that assess orientation, memory, attention, language, and visuospatial abilities. The test takes approximately 10–15 min with a maximum score of 30 points, with lower scores representing higher cognitive impairment. In the current study, the participants were asked to report on the MMSE score in their parent’s last neurological report.

### 2.4. Data Plan Analysis

To examine associations between filial maturity (independent variable), offspring’s resolution of their parent’s disease (dependent variable), and offspring’s well-being (dependent variable), a partial Pearson correlation analysis was conducted, controlling for the adult child’s age. Then, filial maturity’s mediating role between the offspring’s resolution of their parent’s AD diagnosis and the offspring’s well-being was examined with a series of Process models (model no. 4, [24]).

## 3. Results

### Preliminary Analysis

Mean scores for the filial relationships ranged between 3.99 (SD = 0.97) for “parental consideration” and 5.09 (SD = 1.98) for “filial help” (scale 1–7), revealing generally moderate to positive (SD = 0.39) adult child–parent relationships. The total RDQ mean score was 3.27 (scale 1–5), and average well-being was 47.06 (SD = 21.80) (scale 0–100).

Well-being, the dependent variable, was positively correlated with the adult child’s age (r = 0.38, *p* = 0.002), as well as with the parent’s age (r = 0.27, *p* = 0.030). Other demographic variables of the adult child and the parent, such as length of diagnosis, gender, marital status, education level, income level, and whether the parent lived close to the child, were unrelated with well-being (*p* = 0.078 to *p* = 0.980). As the adult child’s age and the parent’s age were highly related (r = 0.49, *p* < 0.001), the former was controlled for in further analyses.

Table 2 presents Pearson correlations between filial maturity, the total RDQ score, and well-being, controlling for the adult child’s age (i.e., partial correlations). Significant negative relationships were found between most of the filial maturity dimensions and the RDQ score, such that better filial maturity was associated with lower resolution of the parent’s diagnosis. Better resolution of the diagnosis, however, was related with better well-being. Nonsignificant relationships were found between the filial maturity dimensions and well-being.

**Figure 1:**
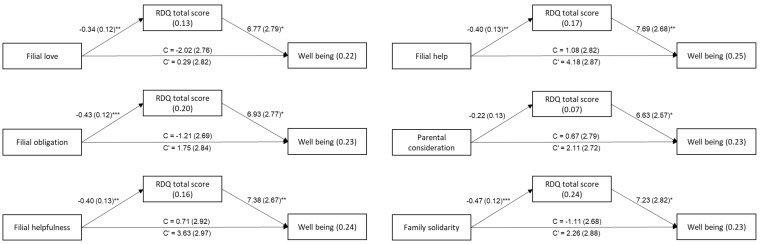
The Mediating Role of RDQ in the Relationship between Filial Maturity and Well-Being. Note. Values on the arrows are B (SE); values within the rectangles are R^2^, C = total effect, C’ = direct effect. * *p* < 0.05, ** *p* < 0.01, *** *p* < 0.001.

Classical mediation theories [25] postulate that the initial C path (IV to DV) must be significant in order to proceed to a mediation analysis. However, current researchers (e.g., [24,26,27]), following Bollen [28], no longer impose the direct relationship as a precondition. Thus, the hypothesized mediation model was calculated. It was calculated with a series of Process models (model no. 4, [24]), due to sample size and the high intercorrelations (r = 0.41 to r = 0.87, *p* < 0.001) among the filial maturity dimensions. Table 3 presents the resulting indirect effects. The results for the mediated relationships appear in Figure 1.

As shown in Table 3 and Figure 1, most mediated relationships were significant, excluding that for “parental consideration”. Apparently, better filial maturity of the offspring is related with lower acceptance of the disease, which is then related with lower well-being.

## 4. Discussion

The first hypothesis that offspring’s resolution of their parent’s diagnosis with AD would be positively correlated with the offspring’s higher well-being was confirmed. Research focusing on family caregivers (spouses or children) has shown that caregivers can experience a vast array of negative emotions, such as sadness, discouragement, loneliness, anger, fatigue, depression, helplessness, and guilt [29]. Furthermore, as the disease progresses, predeath grief as a response to the continuous cognitive, physical, emotional, and behavioral regression of the spouse or parent [2] and the sense of loss of the past and future relationships and hopes, along with the loss of the caregiver’s own freedom [4], affect caregivers’ health and well-being [5]. An optimal resolution of the parent’s loss in the context of the AD diagnosis is an integration of the “new” parent into the previous representations, creating a coherent and nonconflicted set of representations that better represent the new reality and may lead to better well-being [14]. However, when experiencing difficulties with the acceptance of the disease, the offspring becomes preoccupied with negative emotions and thoughts and demonstrates lower well-being. Due to the ambiguous loss experience of offspring of parents with AD, though, it might be that lower well-being is normal for the liminality phase of the ongoing resolution process involving the diagnosis of a person with dementia [2]. Finally, with the current study using a cross-sectional design, it is also possible that the well-being report that related to the previous two weeks may have contributed to their resolution of their parent’s diagnosis with AD result.

The second hypothesis that offspring’s filial maturity would be positively associated with offspring’s resolution of their parent’s diagnosis with AD was disproved. In contrast to the hypothesis, results showed a significant negative association between offspring’s filial maturity and their resolution of their parent’s diagnosis with AD. That is, adult children who were more devoted to their parents and more helpful and felt more loved were less able to accept a diagnosis of their parent with AD.

Filial maturity is a developmental process rooted in childhood and adolescence that enables the adult child to see the parent in a more objective way as a peer and their relationship as more reciprocal [12,13]. Although filial maturity may relieve adult children’s difficulties with the normative aging process of the parent [9,10,11,12,13], it might be highly challenged when a parent is diagnosed with AD, especially as the disease progresses. The discrepancies between highly reciprocal representations of the relationship between the adult child and the parent and the reality of the parent’s mental deterioration as AD progresses could eventually affect the offspring’s ability to come to terms with the parent’s AD diagnosis and delay the integration of the representations of the parent and the relationship they had prior to the diagnosis with those after the parent is diagnosed with AD [14].

It could also be that the ambiguous characteristics of predeath grief [2] and difficulties with accepting the parent’s disease diagnosis influenced participants’ ratings on the filial maturity scale to report a more idealized and reciprocal child–parent relationship, denying the complex reality as part of their coping with the prolonged loss.

The third hypothesis that offspring’s filial maturity would be positively associated with offspring’s higher well-being was not confirmed, as correlations were not significant.

As literature suggests, filial maturity may help adult children to cope effectively with the growing demands of normative caretaking of their aging parent and enhance children’s well-being [9,10,11,12,13]. However. in the case of adult children of parents diagnosed with AD, which is far from normative aging, it might be more complicated.

The WHO-5 Well-Being Index used in the present research asked participants to relate their feelings during the two weeks prior to the report. Hence, well-being of the offspring, most of whom were middle-aged with families, might have been associated with other aspects of their lives, such as marital relationships, family relationships, parenting, etc., rather than the representations of the relationships with the parent that were daily challenged by the AD. Furthermore, it might be that filial maturity has no direct association with well-being of the offspring, but filial maturity associates with well-being through other aspects of having a parent with AD. This is compatible with the current research findings of the mediation role of filial maturity between offspring’s resolution of their parent’s AD diagnosis and offspring’s well-being. According to these findings, when offspring with high filial maturity are characterized by low resolution of their parent’s AD diagnosis, they experience continuous losses of filial relationships due to their parent’s mental and physical deterioration, delaying the integration of the representations of the parent and the relationship they had prior to the diagnosis with those after the parent was diagnosed with AD, leading to low well-being [8,14].

### 4.1. Implications

The current study brings attention to the predeath loss and grief process experienced by offspring of parents diagnosed with AD, broadening prior knowledge that mainly focused on their experience as primary caregivers. As not all offspring are primary caregivers, understanding the prolonged loss they experience and the challenges that affect these adult children enables us to provide the support and care the adult children need in order to have better well-being despite the loss they experience.

Results indicated that coming to terms with their parent’s AD diagnosis was associated with better well-being, but this was difficult for offspring with high filial maturity. Therefore, targeting these individuals within the population of offspring of parents diagnosed with AD may assist them with coping during this challenging period, especially while the disease progresses, and the losses become more ambiguous. It might be that interventions focused on personal resiliency, virtues, and strengths other than familial resources (i.e., filial maturity) could enhance offspring’s ability to resolve their parent’s diagnosis with this disease.

### 4.2. Limitations and Future Studies

Any conclusion regarding causal relationships between variables and generalization are limited due to the cross-sectional design of the study, the relatively small number of participants, and the specific characteristics of the Israeli families constituting the sample (e.g., degree of family intimacy and closeness, religiosity, etc.).

Future research should address other aspects of the offspring’s life, such as marital relationships, work satisfaction, and their parenting of their own children. It should also use interviews to assess offspring’s experience. For a better assessment of the severity of the disease and the functional state of the patient, additional measures such as the Clinical Dementia Rating (CDR) should be used. Research also could examine the contribution of family interventions for adult children’s better resolution of parents’ diagnoses of AD and thus better well-being.

## Figures and Tables

**Table 1:** Background Data for Adult Children and Parents (*N* = 140).

Adult Child		M (SD)/N (%)
Age	24–72	48.99 (10.67)
Number of children	1–6	3.15 (1.16)
Gender	Male	20 (14.3)
	Female	120 (85.7)
Marital status	Single	20 (14.3)
	Married	104 (74.3)
	Divorced/other	16 (11.4)
Education level	High school	20 (14.3)
	Graduate/professional	110 (78.6)
	Other	10 (7.1)
Income level	Below average	24 (17.2)
	Average	58 (41.4)
	Above average	58 (41.4)
**Parent**		**M (SD)/N (%)**
Age	62–92	80.41 (7.06)
Number of years since diagnosis	1–14	4.56 (3.30)
Gender	Male	48 (34.3)
	Female	92 (65.7)
Marital status	Married	64 (45.7)
	Divorced	16 (11.4)
	Widowed	56 (40.0)
	Unknown	4 (2.9)
Lives close to child	Yes	112 (80.0)
Severity of the disease (MMSE score)	Mild (score 21–26)	8 (5.7)
	Moderate (score 15–20)	38 (27.1)
	Moderate-severe (score 10–14)	60 (42.9)
	Severe (score lower than 10)	26 (18.6)
	Unknown	8 (5.7)

**Table 2:** Partial Pearson Correlations between Filial Maturity, Total RDQ Score, and Well-Being (*N* = 140).

Filial Maturity	Filial Love	Filial Obligation	Filial Helpfulness	Filial Help	Parental Consideration	Family Solidarity	RDQ Total Score
RDQ total score *r* (*p*)	−0.33 (0.007)	−0.43 (<0.001)	−0.40 (<0.001)	−0.40 (<0.001)	−0.18 (0.155)	−0.43 (<0.001)	-
Well-being *r* (*p*)	−0.09 (0.468)	−0.06 (0.654)	0.03 (0.809)	0.05 (0.702)	0.03 (0.810)	−0.05 (0.681)	0.30 (0.016)

**Table 3:** Indirect Effects for Well-Being, with RDQ Total Score and Filial Maturity (*N* = 140).

Filial Maturity	B (SE)	95% CI
Filial love	−2.31 (1.32)	−5.23, −0.19
Filial obligation	−2.96 (1.50)	−6.35, −0.52
Filial helpfulness	−2.92 (1.56)	−6.62, −0.47
Filial help	−3.10 (1.64)	−6.91, −0.55
Parental consideration	−1.43 (1.03)	−3.70, −0.45
Family solidarity	−3.37 (1.67)	−7.03, −0.49

Note. The dependent variable is well-being. The mediating variable is RDQ total score. Confidence intervals that do not include zeros are significant.

## Data Availability

The data that support the findings of this study are available on request from the corresponding author. The data are not publicly available due to restrictions, e.g., their containing information that could compromise the privacy of research participants.
